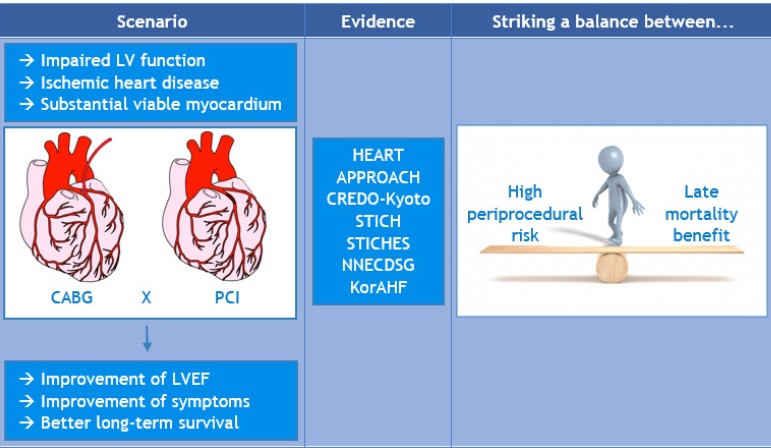# Current Practice of State-of-the-Art Coronary Revascularization in Patients with Heart Failure

**DOI:** 10.21470/1678-9741-2018-0335

**Published:** 2019

**Authors:** Sérgio Costa Rayol, Michel Pompeu Barros Oliveira Sá, Luiz Rafael Pereira Cavalcanti, Felipe Augusto Santos Saragiotto, Roberto Gouvea Silva Diniz, Frederico Browne Correia de Araújo e Sá, Alexandre Motta de Menezes, Ricardo Carvalho Lima

**Affiliations:** 1 Division of Cardiovascular Surgery, Pronto-Socorro Cardiológico de Pernambuco (PROCAPE), Recife, Brazil.; 2 Universidade de Pernambuco (UPE), Recife, PE, Brazil.; 3 Nucleus of Postgraduate Studies and Research in Health Sciences of Faculdade de Ciências Médicas and Instituto de Ciências Biológicas (FCM/ICB), Recife, PE, Brazil.

**Keywords:** Coronary Artery Bypass, Stents, Percutaneous Coronary Intervention, Heart Failure

## Abstract

The best treatment for patients with ischemic heart failure (HF) is still on debate. There is growing evidence that coronary artery bypass graft (CABG) benefits these patients. The current recommendations for revascularization in this context are that CABG is reasonable when it comes to decreasing morbidity and mortality rates for patients with severe left ventricular dysfunction (ejection fraction <35%), and significant coronary artery disease (CAD) and should be considered in patients with operable coronary anatomy, regardless whether or not there is a viable myocardium (class IIb). Percutaneous coronary intervention (PCI) does not have enough data to allow the panels to reach a conclusion. The Korean Acute Heart Failure registry (KorAHF) had its data released recently, showing that patients with acute HF who underwent CABG had lower death rates, more complete revascularization and less adverse outcomes compared with patients treated with PCI. Recent ESC/EACTS guidelines on myocardial revascularization clearly recommended CABG as the first choice of revascularization strategy in patients with multivessel disease and acceptable surgical risk to improve prognosis in this scenario of left ventricular dysfunction. However, a high peri-procedural risk must be compared with the benefit of late mortality, and pros and cons of each strategy (either PCI or CABG) must be weighed in the decision-making process. Spurred on by the publication of the above-mentioned article and the release of new guidelines, we went on to write an overview of the current practice of state-of-the-art coronary revascularization options in patients with HF.

**Table:** 

Abbreviations, acronyms & symbols	
AHF	= Acute heart failure
CABG	= Coronary artery bypass graft
CAD	= Coronary artery disease
DES	= Drug-eluting stent
EACTS	= European Association for Cardio-Thoracic Surgery
ESC	= European Society of Cardiology
HF	= Heart failure
KorAHF	= Korean Acute Heart Failure registry
LAD	= Left anterior descending artery
LM	= Left main
LVEF	= Left ventricular ejection fraction
PCI	= Percutaneous coronary intervention

## INTRODUCTION

Recently, Lee et al.^[1]^ report data from the Korean Acute Heart Failure registry (KorAHF), which is a prospective, multicentre cohort study, aiming to compare coronary artery bypass graft (CABG) and percutaneous coronary intervention (PCI) in patients with acute heart failure (AHF) (propensity score matched).

Some interesting findings were:

1. The rate of death from any cause over 4 years was lower by 40% among patients who underwent CABG than among those who received PCI;
2. In the overall cohort, CABG was associated with lower left ventricular ejection fraction (LVEF) and severe coronary lesions and the characteristics of the patients in the matched cohort were similar to those of CABG group in the overall cohort;
3. The complete revascularization rate, defined as all stenotic main-branch vessels being revascularized, was significantly higher in the CABG group than in the PCI group in the matched cohort;
4. Although the characteristics of the matched population were similar to those of the CABG group in the overall cohort, adverse outcomes were significantly lower in the CABG group than in the PCI group, especially in older patients, those with significant proximal left anterior descending (LAD) artery disease, and those without left main (LM) coronary artery disease (CAD) or chronic total occlusion, the trends favored CABG than PCI.

Spurred on by the publication of the above-mentioned article and the release of new guidelines, we went on to write an overview of the current practice of state-of-the-art coronary revascularization options in patients with HF.

### What are the Current Guidelines for Revascularization in the Context of HF?

Recent European Society of Cardiology (ESC) and European Association for Cardio-Thoracic Surgery (EACTS) guidelines on myocardial revascularization^[2]^ clearly recommended CABG as the first choice of revascularization strategy in patients with multivessel disease and acceptable surgical risk to improve prognosis in this scenario of left ventricular dysfunction (Table 1).

**Table 1:** European guideline-driven recommendations in the context of heart failure.

2018 ESC/EACTS Guidelines on myocardial revascularization Recommendations on revascularizations in patients with chronic heart failure and systolic left ventricular dysfunction (ejection fraction ≤35%)		
Recommendations	Class of recommendation	Level of evidence
In patients with severe left ventricular systolic dysfunction and CAD suitable for intervention, myocardial revascularization is recommended	I	B
CABG is recommended as the first revascularization strategy choice in patients with multivessel disease and acceptable surgical risk	I	B
In patients with one- or two-vessel disease, PCI should be considered as an alternative to CABG when complete revascularization can be achieved	IIa	C
In patients with three-vessel disease, PCI should be considered based on the evaluation by the Heart Team of the patient's coronary anatomy, the expected completeness of revascularization, diabetes status and comorbidities	IIa	C
Left ventricular aneurysmectomy during CABG should be considered in patients with NYHA class III/IV, large left ventricular aneurysm, large thrombus formation, or if the aneurysm is the origin of arrhythmias	IIa	C
Surgical ventricular restoration during CABG may be considered in selected patients treated in centers with expertise	IIb	B

CAD=coronary artery disease; CABG=coronary artery bypass graft; NYHA=New York Heart Association; PCI =percutaneous coronary intervention

According to US guidelines^[3,4]^, revascularization strategies might be beneficial in the context of left ventricular dysfunction (Table 2). CABG surgery would be class of recommendation IIa for those with moderate left ventricular dysfunction and IIb for those with LVEF ≤35% without significant LM CAD. PCI does not have enough data to allow the panels to reach any conclusion nor make any recommendation.

**Table 2:** American guideline-driven recommendations in the context of heart failure.

ACC/AATS/AHA/ASE/ASNC/SCAI/SCCT/STS 2017 Appropriate Use Criteria for Coronary Revascularization in Patients with Stable Ischemic Heart Disease Revascularization to improve survival compared with medical therapy in the anatomic setting of left ventricular dysfunction		
**Recommendations**	**Class of recommendation**	**Level of evidence**
CABG - ejection fraction 35% to 50%	IIa	B
CABG - ejection fraction <35% without significant left main CAD	IIb	B
PCI	Insufficient data	N/A
2013 ACCF/AHA Guideline for the Management of Heart Failure: Executive Summary - Recommendations for Stage C HFpEF - Updated in 2017		
**Recommendations**	**Class of recommendation**	**Level of evidence**
CABG or PCI is indicated for HF patients on GDMT with angina and suitable coronary anatomy, especially significant left main stenosis or left main equivalent	I	C
CABG to improve survival is reasonable in patients with mild to moderate left ventricular systolic dysfunction and significant multivessel CAD or proximal LAD stenosis when viable myocardium is present	IIa	B
CABG is reasonable to improve morbidity and mortality for patients with severe left ventricular dysfunction (ejection fraction <35%) and significant CAD	IIa	B
CABG may be considered in patients with ischemic heart disease, severe left ventricular systolic dysfunction and operable coronary anatomy, regardless of whether a viable myocardium is present	IIb	B

CAD=coronary artery disease; CABG=coronary artery bypass graft; GDMT=guideline-directed medical therapy; LAD=left anterior descending artery; PCI=percutaneous coronary intervention

### What About the Evidence in Other Studies?

One of the first pieces of evidence was the Heart Failure Revascularization (HEART) trial^[5]^, which enrolled 138 patients with HF, CAD and a LVEF ≤35%, who had a substantial volume of viable myocardium with contractile dysfunction, assessed by any standard imaging technique, randomly assigned to a strategy of conservative management *versus* angiography with the intent of PCI or CABG. After a median follow-up of 59 months, there were 25 (37%) deaths in those assigned to the conservative strategy, and 26 (38%) in those assigned to the invasive strategy, 13 (29%) of whom were revascularized. However, this study was underpowered and, further, larger trials were required to settle this issue.

The Alberta Provincial Project for Outcome Assessment in Coronary Heart Disease (APPROACH)^[6]^ compared the outcomes of patients propensity matched to obtain comparable subgroups with CAD and left ventricular dysfunction undergoing CABG (n=718) *versus* PCI (n=718). The analysis identified that CABG was significantly associated with lower rates of repeat revascularization and better survival compared with PCI at 1, 5, 10 and 15 years.

The CREDO-Kyoto PCI/CABG Registry Cohort-2^[7]^ identified 3,584 patients with 3-vessel and/or left main disease of 15,939 patients undergoing first myocardial revascularization, and 908 with LVEF <50%. In both patients with moderate and severe left ventricular systolic dysfunction, the risk of cardiac death after PCI was significantly greater than after CABG. Similarly, the risk of all-cause death tended to be higher after PCI than after CABG in both patients with moderate and severe left ventricular systolic dysfunction. CABG was associated with better 5-year survival outcomes than PCI in patients with LVEF <50% with complex CAD in the era of drug-eluting stents.

In the Surgical Treatment Ischemic Heart Failure (STICH) trial^[8]^, 1,212 patients with CAD and LVEF ≤35% were randomized to medical therapy or CABG. Patients with LM disease were excluded, 17% of patients on medical therapy underwent CABG and 6% of patients underwent PCI by the end of the follow-up period. In the intention-to-treat analysis, all-cause mortality was not significantly lower with CABG than with medical therapy; however, all-cause mortality or hospitalization for cardiovascular causes occurred less frequently among patients undergoing CABG. The results with respect to all other secondary clinical outcomes also favored CABG. In addition, CABG was associated with a reduced risk for the primary outcome (death) in the "as-treated" analysis, which compared the outcomes of 592 patients treated with medical therapy throughout the first year after randomization with those of 620 patients who underwent CABG-either as a consequence of randomization or crossover-and reported significantly lower all-cause mortality in favor of CABG.

In 2016, the authors reported the results of the STICH Extension Study (STICHES)^[9]^, which was conducted to evaluate the long-term effects of CABG in patients with ischemic cardiomyopathy. The rate of death from any cause over 10 years was lower by 16% among patients who underwent CABG in addition to receiving medical therapy than among those who received medical therapy alone. Overall, CABG was associated with an incremental median survival benefit of nearly 18 months and prevention of one death due to any cause for every 14 patients treated and of one death due to a cardiovascular cause for every 11 patients treated. CABG was associated with more favorable results than isolated medical therapy across all clinically relevant long-term outcomes evaluated by the authors. These findings were directionally similar to those reported earlier. The choice between CABG and PCI should be made by the heart team after careful evaluation of the patient's clinical status and coronary anatomy, including SYNTAX Score, comorbidities and expected completeness of revascularization. A specialist in heart failure should be consulted.

Because PCI has become an established treatment option for selected patients with CAD and the STICH trial did not include a PCI arm, some groups sought to assess the comparative effectiveness of CABG *versus* PCI among patients with reduced ventricular function and multivessel CAD. For example, the Northern New England Cardiovascular Disease Study Group (NNECDSG)^[10]^ is a voluntary regional consortium of 7 hospitals in New Hampshire, Vermont, and Maine that provide the majority of PCI and cardiac surgery in the region. The authors examined all patients undergoing primary isolated coronary revascularization from 2004 to 2014. To simulate a real-world STICH-like population, the inclusion and exclusion criteria for the STICH trial were applied. Specifically, were included in the analysis patients who had EF ≤35% and 2- or 3-vessel CAD. The final study cohort was 955 CABG and 718 PCI patients. The median duration of follow-up was 4.3 years. CABG was associated with improved long-term survival compared to PCI after risk adjustment. Although CABG and PCI had similar 30-day mortality rates, CABG was associated with a higher frequency of cerebrovascular accidents and acute kidney injury, whereas PCI was associated with a higher incidence of repeat revascularization. The authors concluded that, among patients with reduced ejection fraction and multivessel disease, CABG was associated with improved long-term survival compared with PCI and should be strongly considered in patients with ischemic cardiomyopathy and multivessel coronary disease.

### Could PCI Provide Comparable Outcomes to CABG in Patients with HF?

Yang et al.^[11]^ enrolled patients with reduced left ventricular systolic function, defined as a LVEF <50%, who had PCI with drug-eluting stent (DES) or CABG from the Cardiovascular Catheterization and Surgery Databases of Samsung Medical Center. There was no statistically significant difference in all-cause death in the follow-up (median duration of 32 months). In the propensity score matching analysis performed in 141 patient pairs, the long-term cumulative mortality rate was not significantly different between the groups. However, the rate of major adverse cardiac and cerebrovascular events was higher in the DES group than the CABG group, which was explained by the higher incidence of repeat revascularization in the DES group.

Bangalore et al.^[12]^ selected patients with multivessel disease and LVEF ≤35% who underwent either PCI with everolimus-eluting stent (n=1063) or CABG (n=1063) with propensity score matching from the New York State Percutaneous Coronary Intervention Reporting System. In the short term, PCI was associated with a lower risk of stroke in comparison with CABG. At long-term follow-up (median of 2.9 years), PCI was associated with a similar risk of death, a higher risk of myocardial infarction, a lower risk of cerebrovascular accident, and a higher risk of repeat revascularization. Completeness of revascularization played a major role in this study, such that, in patients in whom complete revascularization was achieved with PCI, there was no difference in myocardial infarction between PCI and CABG.

### A Word of Caution

Although it seems clear that patients with severe left ventricular dysfunction benefit most from revascularization, nothing is set in stone yet. A high peri-procedural risk must be balanced against late mortality benefit and pros and cons of each strategy (either PCI or CABG) must be weighed up in the decision-making process.

**Table:** 

Authors' roles & responsibilities	
SCR	Drafting the work or revising it critically for important intellectual content; final approval of the version to be published
MPBOS	Substantial contributions to the conception or design of the work; or the acquisition, analysis, or interpretation of data for the work; agreement to be accountable for all aspects of the work in ensuring that questions related to the accuracy or integrity of any part of the work are appropriately investigated and resolved; final approval of the version to be published
LRPC	Drafting the work or revising it critically for important intellectual content; agreement to be accountable for all aspects of the work in ensuring that questions related to the accuracy or integrity of any part of the work are appropriately investigated and resolved; final approval of the version to be published
FASS	Drafting the work or revising it critically for important intellectual content; final approval of the version to be published
RGSD	Drafting the work or revising it critically for important intellectual content; final approval of the version to be published
FBCAS	Drafting the work or revising it critically for important intellectual content; agreement to be accountable for all aspects of the work in ensuring that questions related to the accuracy or integrity of any part of the work are appropriately investigated and resolved; final approval of the version to be published
AMM	Drafting the work or revising it critically for important intellectual content; final approval of the version to be published
RCL	Drafting the work or revising it critically for important intellectual content; final approval of the version to be published

## Figures and Tables

**Figure:**